# Effect of Surface and Interfacial Tension on the Resonance Frequency of Microfluidic Channel Cantilever

**DOI:** 10.3390/s20226459

**Published:** 2020-11-12

**Authors:** Rosmi Abraham, Faheem Khan, Syed A. Bukhari, Qingxia Liu, Thomas Thundat, Hyun-Joong Chung, Chun Il Kim

**Affiliations:** 1 Department of Chemical and Materials Engineering, University of Alberta, Edmonton, AB T6G 2R3, Canada; rosmi@ualberta.ca (R.A.); smanzoor@ualberta.ca (S.A.B.); qingxia2@ualberta.ca (Q.L.); 2 Fourien Inc., Edmonton, AB T6B 2N2, Canada; fkhan@fourien.com; 3 Department of Chemical and Biological Engineering, University at Buffalo, The State University of New York, New York, NY 14260, USA; tgthunda@buffalo.edu; 4 Department of Mechanical Engineering, University of Alberta, Edmonton, AB T6G 1H9, Canada

**Keywords:** microchannel cantilever resonator, vibration resonance frequency, interfacial tension, Euler–Bernoulli beam theory, surface stress

## Abstract

The bending resonance of micro-sized resonators has been utilized to study adsorption of analyte molecules in complex fluids of picogram quantity. Traditionally, the analysis to characterize the resonance frequency has focused solely on the mass change, whereas the effect of interfacial tension of the fluid has been largely neglected. By observing forced vibrations of a microfluidic cantilever filled with a series of alkanes using a laser Doppler vibrometer (LDV), we studied the effect of surface and interfacial tension on the resonance frequency. Here, we incorporated the Young–Laplace equation into the Euler–Bernoulli beam theory to consider extra stress that surface and interface tension exerts on the vibration of the cantilever. Based on the hypothesis that the near-surface region of a continuum is subject to the extra stress, thin surface and interface layers are introduced to our model. The thin layer is subject to an axial force exerted by the extra stress, which in turn affects the transverse vibration of the cantilever. We tested the analytical model by varying the interfacial tension between the silicon nitride microchannel cantilever and the filled alkanes, whose interfacial tension varies with chain length. Compared with the conventional Euler–Bernoulli model, our enhanced model provides a better agreement to the experimental results, shedding light on precision measurements using micro-sized cantilever resonators.

## 1. Introduction

A microcantilever-based sensor driven in dynamic operation mode measures resonance frequencies in various vibrational modes, allowing chemical and biological sensing with extreme precision [1,2,3,4,5]. The resonance frequency can shift by any factors that cause subtle changes in the spring constant or the mass of the microcantilever [4,5,6]. For example, mass and stiffness of an adsorbate can alter those of the resonating microcantilever, resulting in the shift of the resonance frequency in a precisely quantifiable manner [7]; its unprecedented precision level has been crucial in detecting trace amounts of biological analytes, such as toxin, hybridized DNAs, and pathogens [8].

Surface stress can be considered as a measurable macroscopic quantity that arises from the microscopic interactions of adsorbate on surface, surface–environment interaction, or surface reconstruction [9]. As the surface orinterface area-to-volume ratio increases considerably with the reduction of the sizes of the sensors to micron scale, the effects from surface stress become increasingly important [10]. It has been well understood that the surface stress causes a static bending of thin microcantilevers [11]. The effect of surface stress on the natural resonance frequency has been hypothesized since 1975 when Lagowski et al. discovered a strong surface condition dependence on the natural frequency of GaAs crystals with thickness less than 15 µm [12].

Chen et al. [13] and Cherian et al. [14] described the shift in resonance frequency of a cantilever as the combination of increased mass and changed spring constant when a chemical species adsorbs on the surface of the cantilever [10]. Wang et al. emphasized the effect of surface stress on nanomechanical properties of ultra-thin single crystal silicon (SCS) resonators when explaining the effects of thermal treatments and gas adsorption [15]. Hwang et al. performed an experimental study on the correlation between the surface stress exerted by biomolecular interactions and the dynamical response of microcantilevers [16]. A nanomechanical spectrometry study of 100 nm-sized gold nanoparticles (GNPs) and *Escherichia coli* DH5α cells using microcantilever resonators conducted by Malvar et al. revealed that the ignorance of the effect of stiffness can lead to an underestimation of mass by 10% in microcantilevers [17]. A recent project by Stachiv et al. presented an interesting but alternate method to quantify the adsorbate mass by analyzing the changes in the quality (Q) factor without even knowing the position of attachment, stiffness, and surface stress effects [18].

On the theoretical modeling side, McFarland et al. derived an axial beam model with a constant axial force acting on the cantilever based on Lagowski’s hypothesis for explaining the influence of the surface stress on a deformed cantilever [19]. An alternative model was implemented by Ren et al. using a variable axial force in the Euler–Bernoulli governing equation to determine the surface stress effect on the frequency of the microcantilever [20]. Another theoretical explanation for adsorption-induced change in resonance frequency was put forward by Huang et al. by considering the interaction between adsorbates and the cantilever [21]. Along with the experimental evidence, Hwang et al. presented a mechanical beam model for the surface stress driven dynamical response of nanomechanical microcantilevers [16]. In the same year, Dorignac et al. used the fluctuation–dissipation theorem to derive an exact expression for the frequency shift of the nanoscale cantilever placed in a viscous fluid. It was found that the frequency shift was generated by the surface stress due to biomolecular interaction rather than the mass loading [22].

Aforementioned theories based on the hypothesis of surface pressure effect, however, faced numerous criticisms. Gurtin et al. contradicted Lagowski’s hypothesis by stating that within the framework of classical beam theory, strain independent surface stress has no effect on the natural resonance frequency of cantilever beams, but the surface elasticity can change the frequency [10]. In the beam with axial force model, the effective external forces were considerably misinterpreted since the cantilever has a free end to allow deformation or bending to relieve the stress [23]. Wang et al. and Lu et al. analyzed the influence of surface elasticity, pure surface stress, and adsorption induced surface stress on the vibration frequency on the cantilever, stating that only strain dependent surface stress affects the dynamics [23,24]. Strain dependent surface stress explains the external forces that affect the modulus of the cantilever system, whereas strain independent stress is not associated with any generation of strain upon the influence of stress, such as surface tension, interfacial tension, etc.

To address the shortcomings of the previous models, Lachut et al. proposed an alternative approach based on the linear beam elastic theory about unreleased in-plane stress in the vicinity of the supporting clamp [25]. The unreleased stress arises from the clamping restriction for in-plane displacements. The article mentions that the strain independent stress has no role in the resonance frequency of the cantilever [25]. An improved model was employed by Sader et al. in which a cantilever plate model was introduced and considered the effect of cantilever width, which was ignored in Euler–Bernoulli beam theory [26]. However, there was a discrepancy of 2 orders of magnitude in the frequency calculations. Later, Karabalin et al. [27] demonstrated controlled measurements of stress-induced change in cantilever stiffness with theoretical quantification. He concluded that net in-plane stress generated in the immediate vicinity of the supporting clamp in a cantilever by the application of surface stress can alter the resonance frequency to a small but nonnegligible extent. However, the axial force model can predict stress effects in much larger magnitudes because of unspecified and uncontrolled effects [27].

In 2008, He et al. approached the influence of surface stress on resonance bending nanowires by incorporating the generalized Young–Laplace equation into the Euler–Bernoulli beam theory and studying the solution for different boundary condition [28]. He stated that in small deformations, the distributed transverse force resulting from the surface stress along the nanowires longitudinal direction is considered a function of the curvature, and hence he accommodated the strain independent stress and the surface elasticity in the Euler–Bernoulli equation and validated the results [28]. A new model using classical lamination theory was developed by Sohi et al. using the Rayleigh–Ritz method [29]. They accounted for the biaxial curvature variation of the cantilever due to differential surface stress loading, which is the main mechanism responsible for flexural resonance frequency shift. Similarly, Ruz et al. developed a model establishing the size- and vibration mode-dependent nonlinear relationship of change in microcantilever curvature due to surface stress loading and the variation of its resonance frequencies and vibration mode shapes [9]. A similar model by Sohi et al. investigated the modal response of microcantilevers where it relates the bending-extensional mode coupling associated with the flexural vibration modes of microcantilevers with a coupling strength that depends on the microcantilever curvature [30]. An interesting theoretical analysis that enabled the disentangling of the impact delivered by axial force and attached mass on resonant frequency shift of suspended nanomechanical based mass sensors under arbitrary applied axial load was put forth by Stachiv et al. in 2014 [31]. Subsequently, he introduced a similar theoretical investigation of the cantilever beams under an arbitrary value of the axial force vibrating in a specific environment such as a vacuum, air, or a viscous fluid [32].

Apart from surface stress effect, higher order strain gradient theories such as the generalized Cosserat theory of elasticity were considered for more accurate descriptions of micro-mechanical systems, including mathematically tractable formulations and associated analyses of microstructures. The introduction of couple-stress tensor into the models of continuum deformation and analysis of Euler–Bernoulli equation revealed the size-dependent nature of resonant frequencies of the microbeams [33,34,35,36,37,38,39]. The inclusion of a characteristic length parameter (*l*), shear modulus (*µ*), and width of the beam (*A*) in the calculation of Young’s modulus introduced the new effective modulus as $EI^{*}=EI+\;\mu Al^{2}$ and was used to estimate the resonant frequency according to the aforementioned couple stress model [33,34]. The Spencer and Soldatos approach [40] is one of the most widely adopted second gradient models for problems regarding planar beams and plates with meshed microstructures [41,42]. At the same time, the implementation of the nonclassical continuum theories, as in their original forms, has faced formidable challenges especially in characterizing admissible higher forces (e.g., double and triple forces) and their energy couples (Piola-type double and triple stresses) sustained by the higher-order continua [35,36,37,38,39]. One author of this paper, Kim, has contributed to the developments of higher-order gradient models for the mechanics of micro-structured elastic solids subjected to finite plane deformations [43,44,45,46,47,48,49,50].

When the surface effects are not negligible, the bending and vibration analyses of beams can be modeled with the framework of the higher-order gradient-based continuum models with proper dimensional reductions. The dimensional reduction of higher-gradient models leads to the constitutive equations, which share close similarity to those obtained directly from the surface elasticity theory. This is because both approaches essentially result in the same orders of gradient fields once formulated in the form of Euler equilibrium equations.

Though the existing surface elasticity beam models provide comprehensive descriptions in the constitutive modeling level, implementation in an actual problem often results in impractically heavy expressions or even analytically unsolvable expressions [51]. Therefore, it is necessary to devise a prediction model that is simple to implement. A practical target for such an analytical model may be within an allowed error limit of 5%, when the use of heavy computational resources is not preferred.

Although the hypothesis of the surface stress effect on the resonance frequency has been supported by many studies, its practical effect on molecular sensing has been questioned because the resonant vibration of cantilevers dampens in liquid medium, which results in a compromised quality factor of such resonance peaks. In order to address the challenge, microchannel cantilever vibrating in high vacuum environment has been suggested [52,53,54]. The microchannel cantilever, a unique tool to characterize the properties of fluids in picograms of quantity with high sensitivity and selectivity, can operate in two modes [55]. In static mode, the displacement of the beam is converted to the surface stress associated with the adsorption on the cantilever. In dynamic mode, the frequency shift is associated with the surface stress resulting from the adsorption on the beam [56]. Since static mode involves a significant drift in the signal over a large period of time, dynamic mode is preferred for stable long-term operation.

In the present study, we hypothesized that the surface and interface tension of the microchannel cantilever exerts transverse force acting along the longitudinal direction of the deformed beam that modulates the effective modulus of the cantilever system. The presented model is based on the first gradient surface elasticity theory with an assumption of perfect adherence between the fluid substances and the surfaces of the cantilever beam. The microchannel cantilever containing the fluid is considered as a single cantilever system, whose equilibrium shape is in the deformed state; the balance between the internal forces and the surface elasticity is considered [57,58]. Based on the hypothesis, we developed an improved Euler–Bernoulli model. Our main goal is to study the influence of the transverse force along the longitudinal direction, *q*(*x*) in the Euler–Bernoulli equation, on the resonance frequency of the cantilever. This parameter is associated with the second derivative of displacement with respect to position [23,33]. Within these prescriptions, the influences of fluid substances are transmitted through the surfaces of beams, leading to the idealization of surface-influenced beam systems. Our model may be classified as a simplified surface elasticity-based continuum model. When formulating the effective moduli of the beam, $EI^{*}$, the size effects that lead to the frequency shift of the beam are already implied. Then, we tested the hypothesis by monitoring the resonance frequencies of a microchannel cantilever when empty, as well as when filled with a series of alkanes with the number of carbon atoms between five and nine. Our improved Euler–Bernoulli model showed a better agreement with experimental results when compared with the prediction from the conventional model.

## 2. Analytical Model Incorporating Surface Pressure

The classical Euler–Bernoulli beam differential equation for a freely vibrating cantilever with deflection w(x,t) is

(1) $${\left(\rho A\right)}^{*}\frac{\partial^{2}w\left(x,t\right)}{\partial t^{2}}+EI\frac{\partial^{4}w\left(x,t\right)}{\partial x^{4}}=0$$

and its associated natural resonance frequency in the fundamental flexural mode is given by

(2) $$\omega ={\left(\frac{\alpha }{L}\right)}^{2}\sqrt{\frac{EI}{\rho A}}$$

where *L* denotes the cantilever length, *A = b × h* is cross section with *b* and *h* being width and thickness, respectively. *I = bh^3^/12*, *E*, *ρ*, and α = 1.876 are geometric moment of inertia, Young’s modulus, mass density, and mode shape factor for the fundamental mode respectively.

We refine the classical beam model based on the following assumptions:

(1) The length-to-width ratio of the channel is greater than the order of 100.
(2) The amplitude of the beam vibration is smaller than the any beam dimension.
(3) The fluid inside the channel is considered a homogeneous media and incompressible and the beam is a linearly elastic solid.
(4) The deformed beam is in equilibrium considering that all the internal forces are balanced within.
(5) The cross section of beam is uniform over its entire length.

In order to study the surface effects on the natural resonance frequency of microfluidic cantilever, we consider the combined effects of residual surface tension and interfacial tension. The problem is addressed on the basis of a surface layer-based theory taking into account of the origin of interfacial tension and residual surface stress.

**Figure 1:**
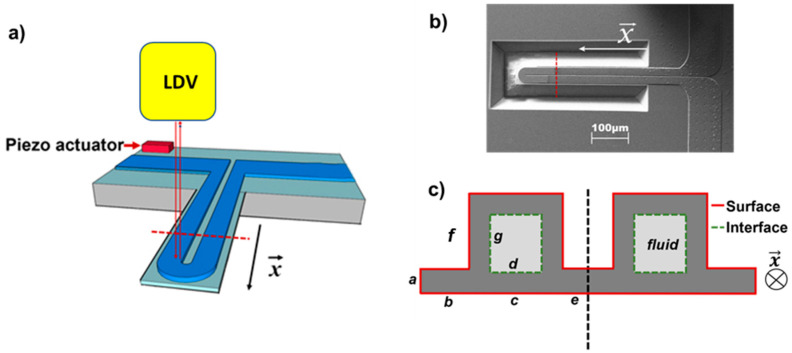
(**a**) Schematic of the experimental system. The microchannel cantilever vibrates in vacuum environment with a pressure below than 1 × 10^−3^ mbar in order to ensure high quality factor resonance peaks. (**b**) Scanning electron microscope (SEM) image from the top of the sensor chip. (**c**) A cross-sectional illustration of the cantilever (dotted red lines in (**a**,**b**)) denoting our definition of interface and surface, as well as the geometric description of the lengths used in our calculation (‘*a*’ to ‘*g*’).

In our study, we use a hollow cannel cantilever filled with fluid. tab 1a shows an overview of experimental setup, and Figure 1b,c shows the top and the cross-sectional view of a microchannel cantilever, respectively.

The surface effects on the natural resonance frequency of the cantilever were studied based on the approach of surface layer model by Wang et al. [23]. Among surface effects, in order to consider both surface elasticity and residual tension on the resonance frequency, the surface elasticity theory of Gurtin et al. [10] was included along with the Young–Laplace equation in the governing differential equation [23]. The microbeam was considered as a sandwich model with thin upper and lower surface layers having near-surface material properties and the bulk material in between. They divided a microbeam into three laminated layers, including two surface layers and a bulk layer with Young’s modulus *E*_1_ and *E* and thickness *h_1_* and *2h*, respectively. The tensile stiffness of the surface layer was denoted as *Es = E*_1_*h*_1_. Letting *h*_1_ approach zero while keeping *Es* as a constant, the surface layer model reduces to the theory of surface elasticity of Gurtin et al. [10] in which the surface has a zero thickness and a surface elastic modulus *Es*.

The effective bending moment of the sandwich beam is expressed as

(*EI*) ^*^ = *EI* + 2*E_s_ah*^2^ (3)

where *I = 2ah^3^/3* and *E* are the inertia moment of the bulk layer and Young’s modulus of bulk layer respectively. Here *a* is the beam width and *E_s_* = *E*_1_*h*_1_ is the tensile stiffness of the surface layer in which *E*_1,_ *h*_1_ are the Young’s modulus and thickness of surface layer, respectively [23].

Our model starts from the similar approach. Since the inner surface of the channel is in contact with the fluid, the system may be modeled as two homogeneous media; i.e., a bulk layer of silicon nitride in contact with a bulk layer of fluid. The Young’s modulus of the channel and the fluid are denoted as *E_H_* and *E_f_*, and they are 180 × 10^9^ Pa and 0.7−0.9 ×10^9^ Pa, respectively [55,59,60].

The effective modulus (EI)* is then given by

(*EI*) ^*^ = *E_H_I_H_* + *E_f_I_f_* (4)

where *I_H_* is the inertia moment of the hollow channel and the *I_f_* is the inertia moment of bulk fluid and are calculated as 5.514 × 10^−23^ m^4^ and 1.8 × 10^−23^ m^4^, respectively. In fact, the effective bending moduli of multilayered beams may be calculated in different ways; for example, those suggested in Zapomel et al. [61], which may result in more accurate predictions. In this novel procedure, measurement of the modulus of suspended micro- or nano-resonators with a deposited thin film was developed by utilizing the Monte Carlo probabilistic method combined with the finite-element method (FEM). In the present study, we adopt the model proposed by Wang et al. [23] based on the theory of surface elasticity for the sake of analytical simplicity and mathematically tractable models.

The peripheral regions of a continuum materials (i.e., atoms or molecules at and near the surface or the interface) have different local environments compared with the bulk region. Thus, the physical properties of these atoms are different; this is the origin of residual surface tension [28]. Here, we denote the residual surface tensions at the surface layer of the silicon nitride channel as *σ* and the interfacial tension between the fluid surface and channel surface as *σ_if_*.

**Figure 2:**
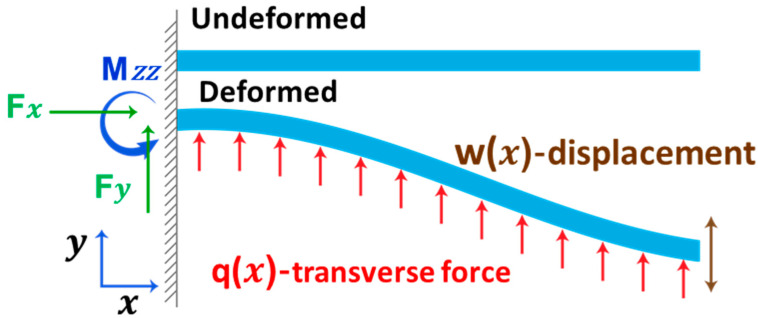
Schematic illustration of the distribution of surface stress on a cantilever under deformation.

In the present study, we adapt the generalized Young–Laplace equation to account the out of plane stresses that are induced from in-plane stresses of the curved interface surfaces. Since the curvature of the beam ($\frac{\partial^{2}w}{\partial x^{2}}$ = 0) vanishes prior to deformation, the residual surface stress has no effect on the bulk [22]. Except for a deformed single beam, the curvature is not zero and the residual surface tension will generate a distributed transverse loading, q(x), along the longitudinal direction on the surface as

(5) $$q\left(x\right)=-pq\frac{\partial^{2}w}{\partial x^{2}}$$

where $\frac{\partial^{2}w}{\partial x^{2}}$ is the local curvature of the beam at *x = x_o_*, *p* is the surface stress, and *q* is the beam width [28]. The distribution of surface stress on a deformed cantilever is shown in Figure 2.

Considering the effects of σ and $\sigma_{if}$ on all four walls of the channel, the Laplace–Young equation on the surfaces yields

(6) $$q_{channel}\left(x\right)=-\left\{2\sigma \left[a+2\left(b+c+g+e\right)\right]\frac{\partial^{2}w}{\partial x^{2}}\right\}$$

and

(7) $$q_{fluid}\left(x\right)=-\left[4\sigma_{if}\left(d+g\right)\frac{\partial^{2}w}{\partial x^{2}}\right]$$

where *a*, *b*, *c*, *g*, and *e* are the characteristic dimension of the hollow channel. When the channel is empty, $\sigma_{if}$ becomes the same as σ. Here, positive $2\sigma \left[a+2\left(b+c+g+e\right)\right]\;and\;4\sigma_{if}\left(d+g\right)]$ are considered as tensile stress and negative are compressive stress. In this case, since all the surfaces are exposed to surface stresses, the situation is complex and compressive stress is taken into consideration as it improves the model value towards the experimental value. Here, the surface stress is not considered as a linear function of surface strain (i.e., sigma = surface moduli × surface strain), which implies that surface stress is not directly contributing to or associated with the curvature and the transverse loading. Instead, it is indirectly coupled with them via Equations (6) and (7).

Thus, effective distributed loading can be formulated as,

(8) $$\begin{array}{cc} q\left(x\right)=q_{channel} & \left(x\right)+q_{fluid}\left(x\right)\\ & =-\left(\left\{2\sigma \left[a+2\left(b+c+g+e\right)\right]\frac{\partial^{2}w}{\partial x^{2}}\right\}+4\sigma_{if}\left(d+g\right)\frac{\partial^{2}w}{\partial x^{2}}\right) \end{array}$$

The equation of motion describing the motion of bending microfluidic channel incorporating the distributed transverse force in the Euler–Bernoulli beam theory is obtained as

(9) $${\left(\rho A\right)}^{*}\frac{\partial^{2}w\left(x,t\right)}{\partial t^{2}}+EI^{*}\frac{\partial^{4}w\left(x,t\right)}{\partial x^{4}}-q\left(x\right)=0$$

and ${\left(\rho A\right)}^{*}$ is the effective density given as

(10) $${\left(\rho A\right)}^{*}=\rho A+\rho_{f}A_{f}$$

in which ρ and $\rho_{f}$ are the densities and *A* and $A_{f}$ are the cross-sectional areas of the channel and fluids respectively. It is seen that both the surface elasticity and residual surface tension affect the dynamic behavior of the beam. The third term in the equation indicates that the influence of residual surface tensions is equivalent to a stretching axial loading $2\sigma \left[a+2\left(b+c+g+e\right)\right]\frac{\partial^{2}w}{\partial x^{2}}$ + $4\sigma_{if}\left(d+g\right)\frac{\partial^{2}w}{\partial x^{2}}$ at the free end of the beam.

For the small harmonic motion, the natural vibration mode of the channel can be decomposed as

(11) w(x,t)=W(x)T(t)

where, W(x) and *T(t)* are normal functions that define the displacement and time, respectively. Plugging Equation (11) into the Euler–Bernoulli equation Equation (9) we obtain,

(12) $$A\;W\left(x\right)\ddot{T}\left(t\right)+B\;W_{xxxx}T\left(t\right)-C\;W_{xx}T\left(t\right)=0$$

where, *A*$={\left(\rho A\right)}^{*}$, *B*= $EI^{*}$ and *C* = $-\left(-\left\{2\sigma \left[a+2\left(b+c+g+e\right)\right]+4\sigma_{if}\left(d+g\right)\right\}\right)$, $W\left(x\right)\ddot{T}\left(t\right)=\frac{\partial^{2}w\left(x,t\right)}{\partial t^{2}}$, $W_{xxxx}T\left(t\right)=\frac{\partial^{4}w\left(x,t\right)}{\partial x^{4}}$ and $W_{xx}T\left(t\right)$ = $\frac{\partial^{2}w\left(x,t\right)}{\partial x^{2}}$.

We consider a cantilever with clamped end at *x = 0* and free end at *x = L*. The boundary conditions for Equation (12) are expressed as

(13) $$w\left(0\right)=0,\;w^{\prime }\left(0\right)=0,\;w^{\prime \prime }\left(L\right)=0,\;w^{\prime \prime \prime }\left(L\right)=0$$

By substituting Equation (12) into Equation (11), Equation (11) becomes

(14) $$r_{1}^{5}+r_{1}r_{2}^{4}+2\;r_{1}^{3}\;r_{2}^{2}\;\cos hr_{1}L\cos r_{2}L+\;r_{1}^{2}\;r_{2}^{3}\;\sin hr_{1}L\sin r_{2}L-r_{2}r_{1}^{4}\sin hr_{1}L\sin r_{2}L=0$$

where

(15) $$r_{1}=\frac{1}{\sqrt{2L}}\sqrt{\left[-s+\sqrt{s^{2}+4\varpi^{2}}\right],}$$

(16) $$r_{2}=\frac{1}{\sqrt{2L}}\sqrt{\left[s+\sqrt{s^{2}+4\varpi^{2}}\right]}$$

and *S =* $\frac{C}{B}$ $L^{2}$; $\varpi^{2}$ *=* $\frac{A}{B}$ $\omega^{2}L^{4}$ in which *S* is the surface effect factor. When *S* approaches zero, the surface effect vanishes, and the above equation yields the same resonance frequency as the classical Euler–Bernoulli equation.

## 3. Estimating Interfacial Tension

The parameter of interfacial tension can be obtained for individual alkanes from the geometric mean equation based on the Owens–Wendt–Rabel–Kaelble method,

(17) $$\gamma_{sl}=\gamma_{s}+\gamma_{l}-2\left(\sqrt{\gamma_{s}{}^{D}\gamma_{l}{}^{D}}+\sqrt{\gamma_{s}{}^{P}\gamma_{l}{}^{P}}\right)$$

where superscripts *D* and *P* represent the dispersive and polar components, $\gamma_{s},\gamma_{l}$ are the surface free energies of solid and liquid, respectively [62]. Since alkanes are considered to be nonpolar, their polar component is zero. The surface energy of silicon nitride is given by 74 mN/m of which the dispersive component is 29 mN/m and the polar component is 45 mN/m [62]. The $\gamma_{l}$, $\gamma_{l}{}^{D}$, and $\gamma_{sl}$ values are summarized in Table 1.

Interfacial tension can contribute either as a positive or a negative stress factor depending on the interaction of fluid with the silicon nitride channel. When the beam height reduces to nanometers or microns, surface effects can be taken into consideration. To further improve the model, the residual surface tension value of silicon nitride is added to the model which is then obtained as

(18) $${\left(\rho A\right)}^{*}\frac{\partial^{2}w\left(x,t\right)}{\partial t^{2}}+EI\frac{\partial^{4}w\left(x,t\right)}{\partial x^{4}}-q\left(x\right)=0$$

and

(19) $$q\left(x\right)=-\{2\sigma \left[a+2\left(b+c+g+e\right)\right]\frac{\partial^{2}w}{\partial x^{2}}+4\sigma_{if}\left(d+g\right)\frac{\partial^{2}w}{\partial x^{2}}\}$$

is the transverse loading term arising from the resultant residual surface tension of silicon nitride and interfacial tension between the fluid and silicon nitride.

## 4. Experimental Details

Alkane series such as pentane, hexane, heptane, octane, and nonane were the test materials due to their absence of polar nature in surface tension, which lessens uncertainty in estimating the interfacial tension. A U-shaped microfluidic channel of 600 μm in length is fabricated on top of the plain cantilever by conventional top-down microfabrication techniques (Figure 1a). The dimensions of the microfluidic channel in Figure 1c are given as *a, b, c, d, e, f*, and *g* where they are 0.90 μm, 10 μm, 32.9 μm, 32 μm, 2.5 μm, 3.9 μm, and 3 μm, respectively.

Prior to loading the alkane samples, the cantilever was cleaned with piranha (in order to remove organic contaminants) and heated on a hot plate at 200 °C for 2 h to drive out any residual moisture. Before loading alkanes, the resonance frequency of the empty channel was noted as a part of the calibration procedure. The cantilever was filled with the alkane samples using syringes by applying a pressure difference where the liquid is pushed through one inlet and the air is pulled out of the other outlet. As a result of the pressure difference, the liquid fills the inside of the channel.

In order to enhance the signal-to-noise ratio (SNR), the cantilever was mechanically excited by an external piezo actuator in the linear regime. The resonance frequency was measured optically using a laser Doppler vibrometer (MSA 500, Polytec, Irvine, CA, USA) with 10 times averaging and was monitored continuously during the experiments. The resonance frequency and quality factor were extracted from the resonance peak. Because the viscosities of alkanes are low and the alkanes are miscible between each other, it was easy to flush and refill the channel to exchange alkane. The alkane inside the channel was completely removed using a vacuum pump and then, cantilever was again placed on the hotplate in order to remove the residues. Prior to loading each sample, the empty cantilever was characterized to determine its resonance frequency and phase. All of the experiments were carried out at a vacuum level of 10^−3^ mbar in order to enhance the quality factor of the system.

## 5. Results and Discussion

**Figure 3:**
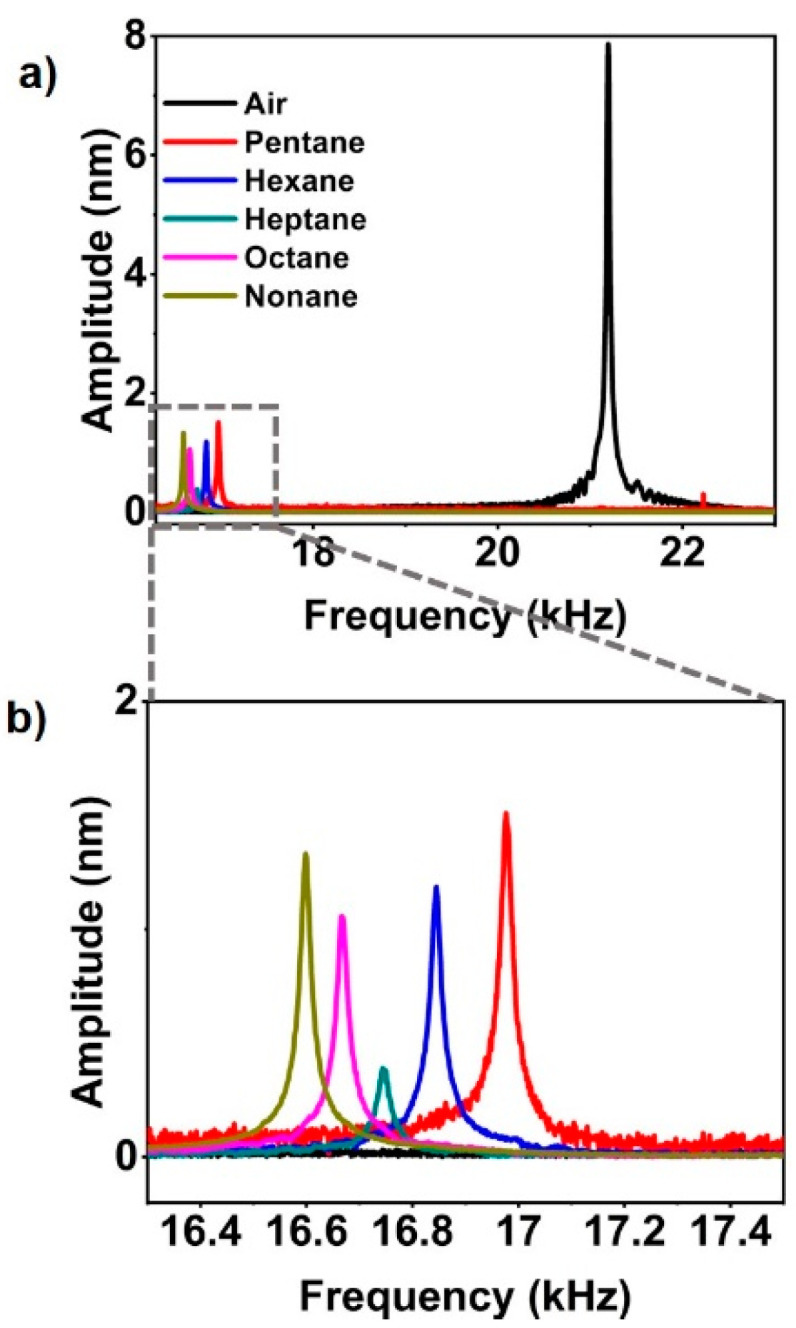
Characterization of resonance frequency of the microchannel cantilever filled with a series of alkanes (**a**) with and (**b**) without the resonance peak of empty channel cantilever.

The filled microchannel cantilevers follow the same dynamics of a plain cantilever because the fluid inside the channel behaves as a homogeneous media. Figure 3 shows the cantilever fundamental frequency as a result of filling of alkanes in the channel. Since the fundamental frequency is dependent on the stiffness and mass, there will be a frequency shift as the cantilever is filled with the alkanes. The frequency of the empty channel cantilever was obtained as 21.19 kHz and the introduction of alkanes caused the resonance frequency of cantilever to shift, varying from 4.20 kHz to 4.59 kHz from the empty channel cantilever frequency. The resonance frequencies of the channel filled with pentane, hexane, heptane, octane, and nonane were obtained as 16.9875 kHz, 16.8672 kHz, 16.7437 kHz, 16.6672 kHz, and 16.5969 kHz, respectively. Once the channel was emptied prior to the filling of the next alkane, it was observed that the frequency increased to 21.19 kHz, which is similar to empty channel frequency. As the density of the alkanes varies from 626 kg/m^3^ to 718 kg/m^3^, it is evident that the shift in frequency is dependent on the mass density addition in the channel. Here, we show that the variation of the frequency shift depends not only on the mass density of the liquids, but also on the transverse force that the liquids cause as the result of exerted interfacial tension.

In order to explain how our enhanced model improves the fitting to the experimental results, we defined nomenclature for the three components in the model as follows: (A) the theoretical prediction of the natural resonance frequency by the conventional model, (B) our new term that includes the effect of the surface and interfacial tension, and (C) the correction factor that takes account for the uncertainty in geometry.

**Figure 4:**
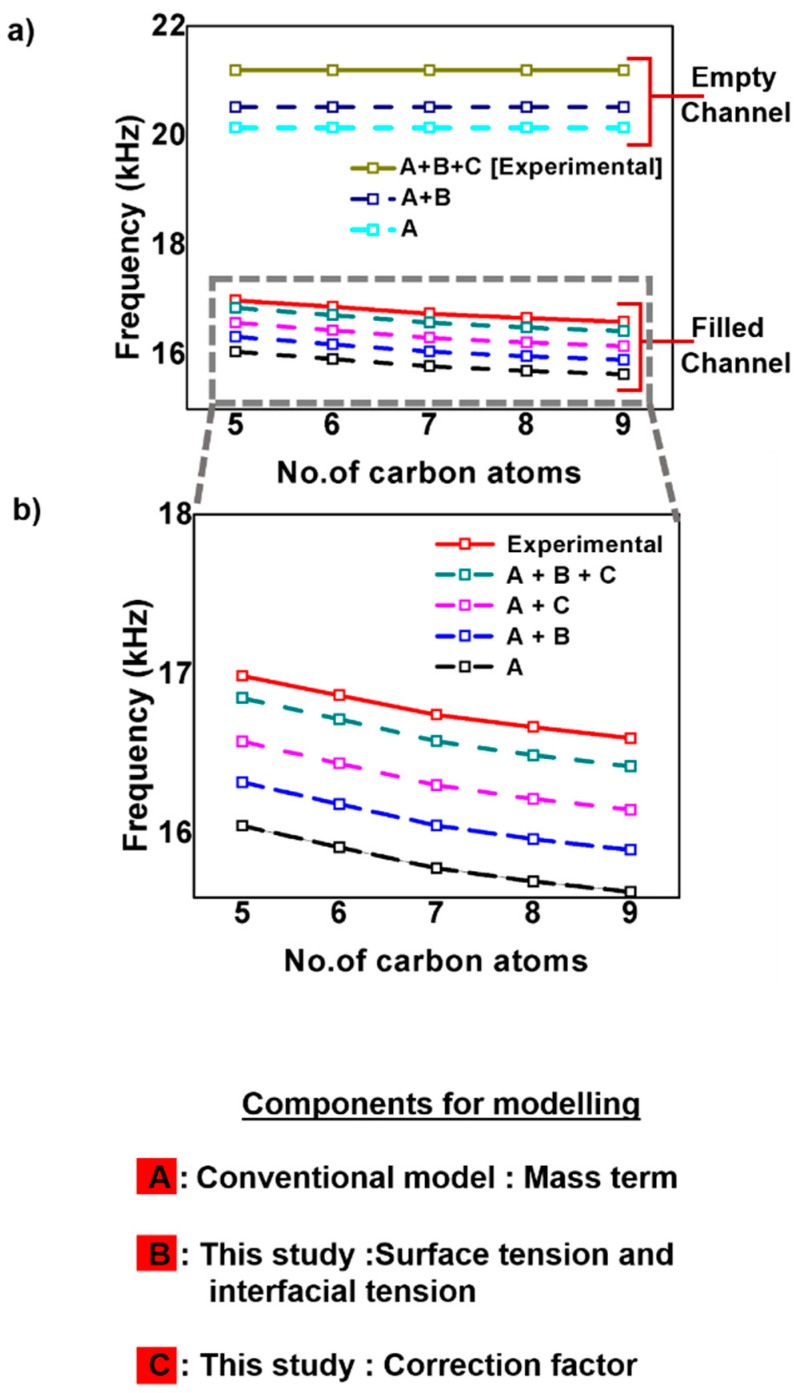
(**a**) Experimentally observed (solid lines) and theoretically predicted (dashed lines) resonance frequencies of the microchannel cantilever. (**b**) Enlarged view of the values from the channel filled with alkane liquids with varying carbon atoms in the chain.

For empty channels, the conventional Euler–Bernoulli model (A) shows a 5.10% discrepancy when compared with the experimental values (Experimental) as shown in Figure 4a. This value (A) is obtained when we substitute *s = 0* in the Equations (15) and (16). When we incorporated the effect of surface and interfacial tension (A+B), the discrepancy decreases to 3.24%. Here, we introduced the correction factor (A+B+C) to reconcile the discrepancy between (A+B) and (Experimental). For the microchannel cantilever that we used in the current study, we obtained the value of the correction factor to be 1.03. Accurate prediction of the resonance frequency of the microfluidic channel can be obtained with the help of finite element analysis of the structure [64,65].

When channels are filled with alkane liquids with carbon numbers between five and nine (Figure 4b), there is a discrepancy of 5.87% between the conventional model (A) and the experimental values (Experimental). Using the surface tension values and the interfacial tension values from Table 1, our modified model (A+B) decreased the discrepancy to 4.19%. Using the correction factor obtained from the empty channel to the filled channel cases, we could achieve good agreement with the experimental results, with an accuracy of 99.03%.

Incorporating the effect of the surface and the interfacial tension into Euler–Bernoulli beam theory in predicting the natural resonance frequency led to a partial reconciliation between the conventional model and the experimental value. In order to further the reconciliation, we introduced the correction factor. Whereas we attribute the physical origin of the correction factor to be an uncertainty in the geometry of fabricated microchannel cantilevers, there is a possibility that the correction factor includes some physics that we overlooked. One observation is that the required correction factor values for empty channel cantilever is clearly different from that of filled cantilevers. This may imply viscoelastic energy dissipation from the enclosed fluid; however, testing the hypothesis will require a series of dedicated theoretical and experimental studies. In addition, it may be of particular mechanical interest to consider slip frictions between the cantilever and liquid medium through an interacting interface. The effects of slip friction may appear in the form of dissipative energy, which was intrinsically limited in the present study where only conservative loads in the sense of virtual work statements are considered.

## 6. Conclusions

In this study, we developed an enhanced Euler–Bernoulli beam model by incorporating Young–Laplace equation to consider transverse force along the longitudinal direction that surface and interface tension exert on the vibration of the cantilever. Our model, which hypothesizes the importance of transverse force on modulating the effective modulus of the microchannel cantilever, emphasizes the importance of surface and interface tension on the natural resonance frequency of the cantilever. We tested the adequacy of our model in a cantilever in its empty channel and alkane-filled channel states. While the conventional Euler–Bernoulli beam model expects the resonance frequency value to be 5.10% and 5.87% lower than the experimental value for empty and filled channel states, the discrepancy value decreases to 3.24% and 4.19%, respectively. It should be noted that our microchannel cantilevers have the wall thickness values of less than a micron, and thus the interfacial tension plays a significant role in the resonance frequency of the cantilever. While our model contributes a partial reconciliation, more studies are needed to elucidate the origin of the remaining discrepancies.

## Figures and Tables

**Table 1:** Surface energy of alkanes and their respective interfacial energy to microchannel cantilever walls made of silicon nitride (estimated) [63]. Note, alkanes can be considered as purely nonpolar (i.e., $\gamma_{l}\approx \gamma_{l}{}^{D}$).

Alkane	$\gamma_{l}(\mathrm{mN}/m)$	$\gamma_{l}{}^{D}(\mathrm{mN}/m)$	$\gamma_{sl}(\mathrm{mN}/m)$
Pentane	15.82	15.82	46.9817
Hexane	18.4	18.4	46.2004
Heptane	19.9	19.9	45.8542
Octane	21.3	21.3	45.5929
Nonane	23	23	45.3473

## Data Availability

The data that support the findings of this study are available from the corresponding author upon reasonable request.
